# High inbreeding, limited recombination and divergent evolutionary patterns between two sympatric morel species in China

**DOI:** 10.1038/srep22434

**Published:** 2016-03-01

**Authors:** Xi-Hui Du, Qi Zhao, Jianping Xu, Zhu L. Yang

**Affiliations:** 1 Key Laboratory for Plant Diversity and Biogeography of East Asia, Kunming Institute of Botany, Chinese Academy of Sciences, Kunming, Yunnan 650201, China; 2 Department of Biology, McMaster University, Hamilton, ON L8S 4K1, Canada

## Abstract

As highly prized, popular mushrooms, morels are widely distributed in the northern hemisphere, with China as a modern centre of speciation and diversity. Overharvesting of morels has caused concern over how to effectively preserve their biological and genetic diversity. However, little is known about their population biology and life cycle. In this study, we selected two sympatric phylogenetic species, *Mel-*13 (124 collections from 11 geographical locations) and *Morchella eohespera* (156 collections from 14 geographical locations), using fragments of 4 DNA sequences, to analyse their genetic structure. Our results indicated significant differentiation among geographic locations in both species, whereas no obvious correlation between genetic and geographic distance was identified in either species. *M. eohespera* exhibited a predominantly clonal population structure with limited recombination detected in only 1 of the 14 geographic locations. In contrast, relatively frequent recombination was identified in 6 of the 11 geographic locations of *Mel-*13. Our analysis indicated that the sympatric species *Mel-*13 and *M. eohespera* might have divergent evolutionary patterns, with the former showing signatures of recent population expansion and the latter being relatively stable. Interestingly, we found no heterozygosity but strong evidence for genealogical incongruence, indicating a high level of inbreeding and hybridisation among morel species.

Morels are among the world’s most prized edible fungi, and are collected by both fungal enthusiasts and food specialists due to their unique culinary flavour and rarity. Although cultivation of *Morchella rufobrunnea*^1^ and *M. importuna*^2^ has been achieved separately in the USA and China, morels collected from the wild still dominate the markets. To meet the demand created by their growing popularity, wild morels are heavily harvested and traded extensively in China, India, Turkey, Mexico and the USA^3^. As with many other wild gourmet mushrooms, the loss and fragmentation of habitat and exploitative over-harvesting have created significant challenges for the management of natural populations^3^,^4^. A better understanding of their population biology is needed to facilitate the development of conservation strategies to maintain wild populations of morels.

Fungal mating systems play important roles in shaping the genetic structure of fungal populations^5^,^6^,^7^,^8^,^9^,^10^, yet little is known about the life cycle and reproductive systems of morels. Such knowledge is critical to understanding their population dynamics and the effects of habitat fragmentation and harvesting practices on their genetic structure. Determining the patterns of genetic variation using molecular markers will provide information on the population genetics of morels, which can also be used to infer their life cycle and mode of reproduction. Several studies have indicated that species in the Elata and Esculenta clades of *Morchella* might be heterothallic and could outcross in nature^11^,^12^,^13^,^14^,^15^. However, the reproductive modes of *Morchella* species are still not clearly known, and it is unknown whether inbreeding or clonality exists in the life cycle of morels. Dalgleish and Jacobson^11^ and Pagliaccia *et al.*^14^ emphasised the importance of further research to resolve important aspects of the morel life cycle regarding heterokaryosis and inbreeding potential. To address these issues, more extensive sampling and the use of highly polymorphic co-dominant molecular markers are needed to detect genetic variation within and between populations and to infer critical issues regarding the mating system of morels.

China is known for its complex geological and ecological diversity. Our recent study identified China as the modern species diversity centre of *Morchella*^16^. Interestingly, several closely related species are sympatric (unpublished data). The high species diversity and sympatric species distribution pattern make China an ideal region in which to analyse and compare the genetic diversity, patterns of genetic variation and modes of reproduction of morels. In this study, we analysed and compared the genetic structure of two sympatric morel species (*Mel-*13 *Morchella* sp. proved as the valid phylogenetic species^16^ and *M. eohespera*) from China using multi-locus sequence data. We aimed to address the following questions. (1) What is the spatial genetic structure, the level of genetic diversity within and among populations and the degree of population differentiation within each of the two sympatric species? (2) Are their evolutionary histories similar to or divergent from each other? (3) What are their potential modes of reproduction? Are their reproductive modes dominated by clonality, recombination or a mixture of both? Our findings will help in understanding the reproductive modes and the distributional patterns of genetic variability in natural populations of morels, which is essential for developing effective strategies for the management and conservation of these two morel species.

## Results

### Sequence variation within four sequenced fragments of Mel-13 and *M. eohespera*

We successfully obtained all of the sequences from four DNA fragments for 280 samples (124 samples of *Mel-*13 and 156 samples of *M. eohespera*). No heterozygosity was observed for any site in the four genetic makers of both species.

For *Mel-*13, the observed polymorphic sites (percentage)/total aligned nucleotide sites for B1, F1, F2 and ITS rDNA were 1 (0.16%)/638, 6 (0.85%)/706, 4 (0.59%)/673 and 11 (0.17%)/666, respectively, separating the 124 collections into 2, 8, 4 and 7 sequence types or haplotypes, respectively. For *M. eohespera*, the observed polymorphic sites (percentage)/total aligned nucleotide sites for B1, F1, F2 and ITS were 5 (0.78%)/638, 8 (1.24%)/644, 10 (1.5%)/670 and 16 (2.3%)/690, respectively, separating the 124 collections into 5, 6, 8 and 15 sequence types or haplotypes, respectively.

### Population Structure

#### *Mel*-13

**Figure 1:**
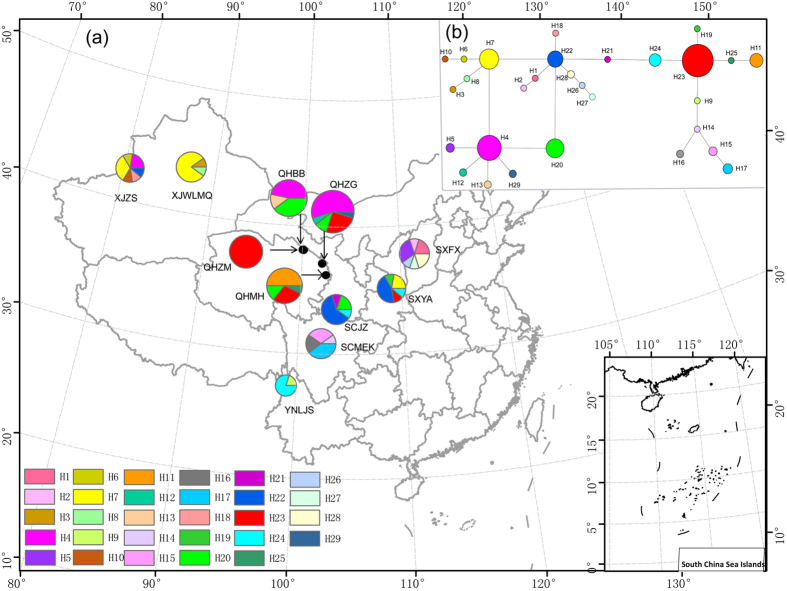
(**a**) Geographic location of the 14 populations of *Mel*-13 analyzed in the present study and the distribution of the 29 haplotypes detected (see Table 1 for population codes). Pie chart size corresponds to the sample size of each population. (**b**) Median joining phylogenetic networks among haplotypes of Mel-13. Maps were generated using ArcView GIS 3.2 (ESRI, Redlands, CA, USA) to estimate the distance between genets and locations.

Twenty-two variable sites were found among the 2682 aligned nucleotide sites for the combined four-gene dataset, the variable site proportion of which was 0.82%. Of the 29 haplotypes detected in this species (Table 1), 23 (79.3%) were private haplotypes, each found in only one population. Most of these private haplotypes were represented by one sample each. The remaining six haplotypes were shared by collections from two or more geographic locations. Haplotypes H7, H14, H22 and H23 were widely distributed. The SXFS population had the highest haploid diversity and nucleotide diversity. Interestingly, none of the four common haplotypes (H7, H14, H22 and H23) were found in this population. The median joining network showed that the remaining 25 haplotypes were linked to these 4 main haplotypes in a ‘star-like’ network. Except for the QHZM population, which had only one haplotype (H23), each population had at least two haplotypes (Fig. 1).

The molecular genetic diversity indices, H_d_ and π, for each population are summarised in Table 1. The highest haplotype diversity (H_d_ = 0.889) was detected in the XJZS and SXFS populations, with nucleotide diversity (π) of 0.00083 and 0.0015, respectively. The QHZM population had the lowest H_d_ (0), with only one haplotype (H23).

Nei’s genetic distance between pairwise members of the population ranged from 0.009 (between QHBB and QHZG) to 0.663 (between SCJZ and SCMEK) (Table S1). The estimated mean gene flow, Nm, among populations was 0.53. Based on a MultiLocus analysis, the 11 geographic locations showed significant genetic differentiation with a value of 0.4877, significantly larger than the randomised datasets (*p* < 0.001).

The SAMOVA analysis showed that the Φ_CT_ value was highest when K = 9. However, as it made little difference when the 11 populations were defined as 9 groups, we do not define the groups in *Mel-*13 here.

The AMOVA revealed that 49% of genetic variation was within populations and 51% between populations (Table 2), and the results were statistically significant. Mantel tests (Figure S1) were conducted to determine whether the observed genetic differentiation was related to geographical and altitudinal differences. A positive correlation was found for geographical distance (*p* = 0.2) and a negative correlation for altitude (*p* = 0.19), although the correlations were not statistically significant.

The unimodal graphs illustrating the mismatch distribution analyses (Figure S3) for *Mel-*13 indicated a sudden population expansion, inferred by the near-perfect fit between the observed and the expected mismatch distribution, and supported by non-significant SSD and HRI statistics (Table 3). The star-like pattern exhibited by the neighbouring joining network (Fig. 1) was also characteristic of recent population expansion. However, the neutrality tests produced insignificant positive values for Fu’s FS and Tajima’s D statistics, as expected for stationary populations (Table 3).

#### M. eohespera

**Figure 2:**
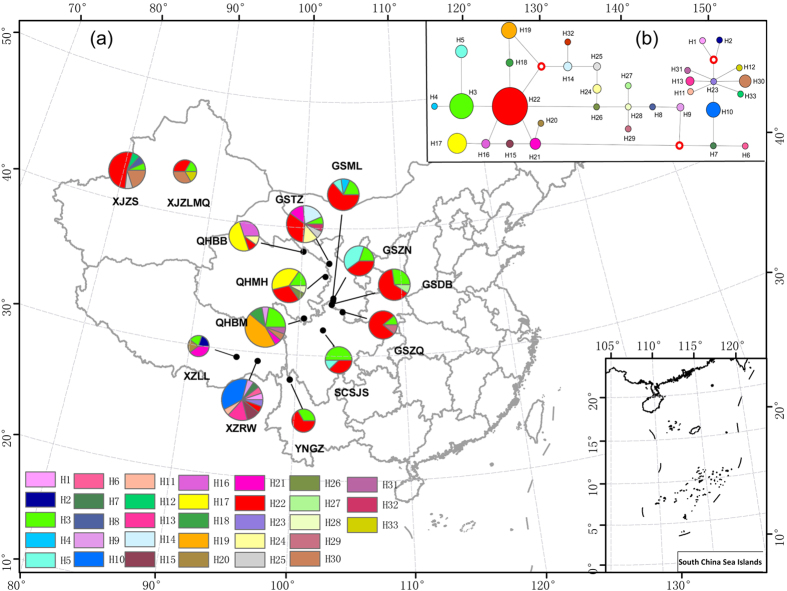
(**a**) Geographic location of the 14 populations of *M. eohespera* analyzed in the present study and the distribution of the 33 haplotypes detected (see Table 1 for population codes). Pie chart size corresponds to the sample size of each population. (**b**) Median joining phylogenetic networks among haplotypes of *M. eohespera*. Maps were generated using ArcView GIS 3.2 (ESRI, Redlands, CA, USA) to estimate the distance between genets and locations.

Among the 2642 aligned nucleotide sites in the combined four-gene dataset, 39 variable sites were found, thus the proportion of variable sites was 1.48%. Of the 33 haplotypes detected in this species, 17 (51.5%) were private haplotypes, each found in only one population (Table 1). H22 was the most abundant haplotype, distributed in 12 of the 14 geographic locations. XZRW harboured the highest number of haplotypes among all of the populations, and also had the richest and most widely distributed haplotype, H22. All populations had two or more haplotypes each. The median-joining haplotype network of these 33 haplotypes is shown in Fig. 2.

The molecular genetic diversity indices, H_d_ and π, for each population are summarised in Table 1. The XZLL population had the highest haplotype diversity (H_d_ = 0.9) and XZRW had the highest number of haplotypes (n = 10, H_d_ = 0.867). The XJWLMQ and GSZQ populations had the highest nucleotide diversity (π, 0.00318) and the lowest H_d_ (0.417), respectively.

Nei’s genetic distance between pairwise members of the population ranged from 0.001 (between GSDB and GSZQ) to 0.254 (between QHBB and XZRW) (Table S2). The average Nei’s genetic distance among populations was 0.0552. The estimated mean gene flow, Nm, among populations was 0.27. A MultiLocus analysis of the 14 populations showed significant genetic differentiation with a value of 0.3675, significantly larger than the randomised datasets (*p* < 0.001).

In the SAMOVA analysis with K = 2, two groups were well defined and the Φ_CT_ value was the highest, corresponding to the XZRW population as one group and the remaining populations as the other. Based on the groups defined by SAMOVA, the AMOVA analysis revealed that 35% of genetic variation was within populations, 7% between populations and 58% between groups (Table 2), all of which were statistically significant. Mantel tests were conducted to determine whether the observed genetic differentiation was related to their geographical and altitudinal differences. Negative correlations were found for both geographical distance (*p* = 0.25) and altitude (*p* = 0.52), but they were not statistically significant (Figure S2).

The multimodal graphs from the mismatch distribution analyses (Figure S4) for *M. eohespera* indicated stationary populations, and this was supported by the non-significant positive value of Fu’s FS and Tajima’s D generated in the neutrality tests. However, the SSD and HRI statistics were non-significant, which is unexpected for stationary populations (Table 3).

### Evidence for clonality and recombination

We performed multilocus linkage disequilibrium statistical tests, including the I_A_ and phylogenetic incompatibility tests, on our data using two methods. First, to detect whether recombination existed within each gene fragment, we conducted these tests respectively in B2, F1, F2 and ITS rDNA fragments for the total samples of *Mel-*13 and *M. eohespera*. Each variable nucleotide site was treated as a locus and different nucleotides at the same site were viewed as different alleles. The results (Table 4) showed no recombination in the F2 and ITS DNA fragments in *Mel-*13, except for the B2 gene fragment, which was excluded because only one polymorphic site was detected. However, the phylogenetic incompatibility test identified evidence of recombination within the F1 gene fragment, which was indicative of limited recombination. In *M. eohespera*, no recombination was detected in the B2, F1 and F2 gene fragments, but limited recombination was found in the ITS region according to the rBarD value, which was not significantly different from the null hypothesis.

Second, it is easy to bring the linkage disequilibrium within a gene fragment to that between DNA fragments when using nucleotide variation within and among genes. Thus, to examine the associations between the alleles of the four genes, we defined four DNA fragments as four loci and used the allelic data from each locus to analyse the relationships among the alleles from different loci. Based on this revised data, multilocus linkage disequilibrium statistical tests were conducted for two sample types: the total samples and samples in each population (Table 5).

For the total samples in both *Mel-*13 and *M. eohespera*, our analyses revealed that the rBarD values were significantly different from the null hypothesis of random recombination, but the phylogenetic incompatibility test identified limited evidence of recombination. These results indicate that limited recombination partly contributed to differentiation in both species.

Of the 11 populations within *Mel-*13, the rBarD and PrC values showed significant evidence of recombination in the SXFS population; five populations (XJZS, XJWLMQ, SXYA, SCJZ and YNLJS) were indicative of limited recombination by the PrC values; and the other five (QHZM, QHMH, QHBB, QHZG and SCMEK, accounting for 45.5%) showed no evidence of recombination. Of the fourteen populations in *M. eohespera*, the PrC value indicated limited recombination only in the XZRW population. Thus, *Mel-*13 showed more evidence of recombination than *M. eohespera*.

### Hybridisation or horizontal gene transfer and sympatric distribution

**Figure 3:**
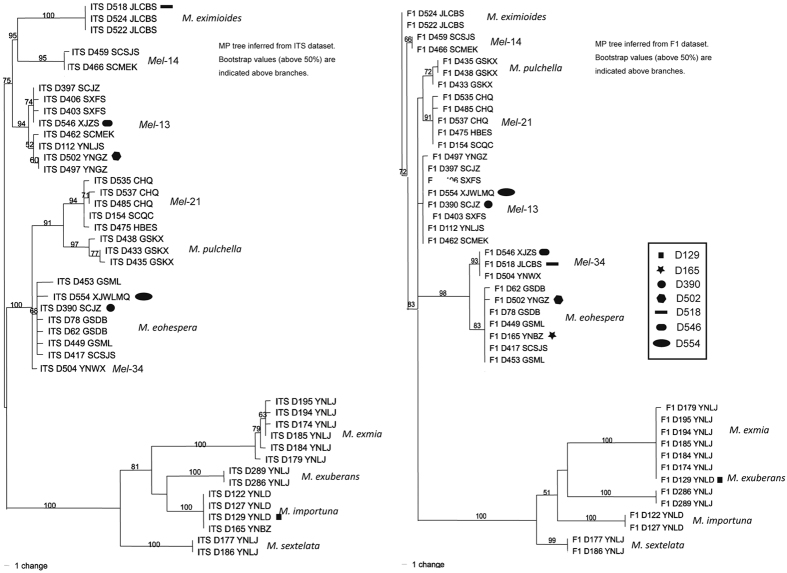
Maximum-parsimony trees showing the relationships among 43 collections in eleven species of Elata Clade. The tree for the left and the right correspond to ITS and F1, respectively. These seven collections probably experienced hybridization or horizontal gene transfer, which were indicated by seven different symbols shown on the trees, respectively.

During the preliminary screening of the 496 samples collected in China using sequences from the four gene fragments, three of the markers showed largely consistent gene genealogical patterns, but that of the F1 fragment was different. Among the 496 strains, 7 showed evidence of hybridisation or horizontal gene transfer: D129, D165, D390, D502, D518, D546 and D554 (Fig. 3). According to the individual phylogenetic analysis of ITS, B2 and F2, D129 and D165 were nested within *M. importuna*, D502 and D546 nested within *Mel-*13, D518 nested within *M. eximioides*, D390 and D554 nested within *M. eohespera*. However, the results based on the F1 gene fragment showed that D129 was nested within *M. eximia*, D390 and D554 were nested within *Mel-*13, D165 and D502 were nested within *M. eohespera* and D518 and D546 were nested within *Mel-*34 (Fig. 3; ITS and 43 collections were used as the representatives). To confirm that these collections did have the unusual sequences, we re-extracted their DNA and sequenced the four loci again. All sequences were found to be the same as those obtained in the initial sequencing. Our results suggest that the special alleles in F1 among these six species were probably due to recent hybridisation or horizontal gene transfer. Analyses of additional markers should help to reveal the extent of hybridisation among the lineages. Due to the conflicting findings for the F1 DNA fragments and the other potential hybridisation individuals, they were not included in the population studies.

Sympatric distribution was also found to be common in *Morchella*. For example, the 19 samples collected from the SCMEK population (found on a small mountain) belonged to three species, *Mel-*13 (n = 10), *Mel-*14 (n = 7) and *M. eohespera* (n = 2). Similarly, in the HBES population, 11 samples collected less than 30 m apart were found to represent 3 species, *M. eohespera* (n = 1), *Mel-*21 (n = 9) and *M. pulchella* (n = 1). In the SCSJS population, 9 samples collected less than 50 m apart represented 2 species, *Mel-*14 (n = 1) and *M. eohespera* (n = 8). In the SCQC population, 10 samples collected from less than 100 m apart represented two species, *Mel-*21(n = 8) and *M. pulchella* (n = 2). Their sympatric distribution affords plenty of opportunities for hybridisation among the lineages.

## Discussion

This is the first study conducted on the population biology of two closely related sympatric morels species. Both species showed rich and divergent genetic diversity. The ITS region and the B2 gene fragment, respectively, harboured the most polymorphic sites and the fewest polymorphic sites in both species and all four DNA fragments showed more variation in *M. eohespera* than in *Mel-*13. Some studies have suggested that increases in genetic polymorphism, mutation and recombination might ensure higher levels of genetic diversity, thus providing greater potential for genetic adaptation in changing environments^17^,^18^,^19^,^20^,^21^. No heterozygosity was observed in either species, even for the ITS region, which is known to be multi-copied within the nuclear ribosomal gene cluster. The lack of heterozygosity suggests that the fruiting bodies derived from haploid mycelia and that these four DNA fragments represent single copy gene markers. Yoon *et al.*^22^ indicated that species in the *M. esculenta* complex (Esculenta Clade) were haploid due to no heterozygosity found, as similar results were observed in *Mel-*13 and *M. eohespera* (Elata Clade). It is presumed that selfing might be very common in these morel species or they were homothallic and their fruiting bodies were developed from haploid mycelia (see the latter discussion).

Significant differentiation was observed among geographic locations within *Mel-*13 and *M. eohespera*. However, no correlation was detected between genetic distance, geographic distance and altitude (Figure S1 and Figure S2). Although geographic separation has been shown to be an important contributor to genetic differentiation in many species, environmental factors can also play a significant role^23^,^24^. For example, habitat fragmentation has been found to alter the structure, distribution and functioning of natural ecosystems^25^, and to increase population genetic divergence, inbreeding or selfing and reduce gene flow^26^,^27^,^28^. The rich private haplotypes observed in *Mel-*13 (79.3%) and *M. eohespera* (51.5%) indicated potential multiple habitat fragmentation events. Considering that the speciation time of *Mel-*13 and *M. eohespera* has been estimated at around 2.855 Mya and 0.443 Mya respectively^16^, these habitat fragmentation events were likely to have been influenced by the Quaternary Glaciation. Moreover, mating patterns tended to shift towards an increased rate of selfings buffering the genetic effects of habitat fragmentation^29^. Consistent with the prediction, we found clear evidence of clonality/selfing in *Mel-*13 and *M. eohespera*.

Although *Mel-*13 and *M. eohespera* have sympatric distributions in southern China, our results indicated that they probably had different evolutionary histories. The mismatch distribution analysis and the median joining network indicated that *Mel-*13 probably experienced a sudden population bottleneck followed by expansion, while *M. eohespera* did not. The speciation time for *Mel-*13 has been estimated at about 2.855 Mya. During its evolutionary history, the climatic oscillations of the Quaternary led to repeated drastic environmental changes (<2.0 Mya)^30^,^31^. These changes probably promoted the sudden expansion of *Mel-*13 and stimulated the emergence of very rich private haplotypes (79.3%) and more frequent recombination detected in 54.5% of the *Mel-*13 population, which may have helped it to survive the extensive environmental changes and habitat fragmentation. Different genotypes may display different fitness in different environments, so the highly localised genetic diversity could have accelerated adaptation to heterogeneous and changing biotic and abiotic environments^32^,^33^.

It has been estimated that *M. eohespera* emerged around 0.443 Mya, during the Pleistocene^16^. Four major glaciations have been proposed in the Qinghai-Tibet Plateau during the Quaternary, and they became progressively less extensive after the largest Naynayxungla Glaciation (c.0.72–0.5 Mya)^34^,^35^,^36^. It is thus unlikely that the last glaciation had a strong influence on *M. eohespera*, which partly explains why fewer private haplotypes (51.5%) were found in *M. eohespera* than in *Mel-*13, and why limited recombination was identified only in the XZRW population. Our results suggested that the XZRW population was the centre of diversity of *M. eohespera* in China because this population possessed the most haplotypes (n = 10), including the most broadly distributed haplotype, H22, and the SAMOVA analysis separated it from the other populations.

The reproduction mode of morels has been debated by various authors. Studies have indicated that collections identified as *M. esculenta*, *M. deliciosa* and *M. snyderi* are heterothallic and could be outcrossed in the laboratory^12^,^14^,^37^,^38^. However, Dalgleish and Jacobson^11^ and Pagliaccia^14^ raised the possibility that some populations from the US probably incorrectly reported as *M. esculenta* and some individuals of *M. snyderi* were clonal or had selfing as their dominant reproduction mode.

Our current results identified limited recombination within the four combined loci of the total samples for both species, and within the F1 gene fragment of *Mel-*13 and the ITS region of *M. eohespera*, however, no evidence of recombination was found in the other DNA fragments in either species (Table 4). In *Mel-*13, 45.5% of the populations (QHZM, QHMH, QHBB, QHZG and SCMEK) appeared to be asexual, and five populations (XJZS, XJWLMQ, SXYA, SCJZ and YNLJS) appeared to have limited recombination. Notably, only one population (SXFS) showed evidence of recombination according to both rBarD and PrC values, and this population had the highest haploid diversity and nucleotide diversity. Among the 14 populations in *M. eohespera*, 13 showed no evidence of recombination, consistent with asexual reproduction. Only the XZRW population showed limited recombination according to the PrC value. Similar to *Mel-*13, XZRW, the only population where recombination was detected in *M. eohespera*, also had high haploid and nucleotide diversity. Interestingly, two other populations, XJWLMQ and XZLL, in *M. eohespera* also had high haploid and nucleotide diversity, but no recombination was identified in them.

Taylor *et al.*^39^ proposed that clonality and recombination can be temporally or spatially separated in a single fungal species. Indeed, many fungal populations have been shown to contain signatures of both recombination and clonal expansion of a few genotypes^40^,^41^,^42^,^43^,^44^. Our analysis revealed a general picture of admixture. A predominantly clonal reproductive strategy with limited recombination was inferred for *M. eohespera*. The results were supported not only by the results of a multilocus linkage disequilibrium analysis, but also by the identification of a few over-represented genotypes (H3 and H22), consistent with clonal expansion. This strategy might facilitate the dispersal of individuals colonising a new habitat, as proposed by Dalgleish and Jacobson^11^ and documented in mycorrhizal basidiomycetes^45^,^46^,^47^. Clonal reproductive modes were also prevalent in *Mel-*13, but recombination events were much more frequent in *Mel-*13 than in *M. eohespera*, which probably stimulated the increase in genetic diversity that might have enhanced the adaptation of some populations to hostile environmental and climate changes. The different populations in *Mel-*13 may have had different reproductive strategies, whereas most populations in *M. eohespera* possesses clonal reproductive mode, and the levels of genotypic diversity were at least partly associated with the level of recombination.

It is not known how variation in reproductive mode across morel populations is controlled, or whether these distinct populations use sexual and/or asexual reproductive modes to regulate gene flow, select more rapidly adaptive genotypes and respond to heterogeneous environments. Extensive sampling and laboratory mating assays would help to gain a full understanding of the mating behaviour and determine the relative contribution of asexual versus sexual reproductive modes in morels. We cannot reject the possibility that both species are heterothallic, and speculate that the unknown mating system may also facilitate outcrossing considering the high level of genetic variation in the populations of both species.

If the life cycles of *Mel-*13 and *M. eohespera* were heterothallic, the lack of heterozygosity in any of the samples from any of the four DNA fragments would suggest that inbreeding must be highly prevalent. The presence of hybridisation, as suggested by the gene genealogical incongruence, indicates that mating must have occurred in nature. If this were true, the lack of heterozygosity would indicate that inbreeding must have been the predominant mode of sexual reproduction.

At least five species of *Morchella* were found to be sympatric in our study, but whether there is reproductive isolation between them and what’s the isolation degree is unknown. Some studies have proposed greater reproductive isolation is involved in sympatric species than allopatric ones^48^,^49^. However, Le Gac and Giraud^50^ indicated no significant difference in the degree of reproductive isolation between sympatric and allopatric pairs of ascomycota, and found that many pairs of closely related sympatric species in ascomycota were not isolated by strong premating barriers. This is supported by our observation that crossing experiments on these morel species under laboratory conditions showed little evidence of reproductive isolation (unpublished data). Le Gac and Giraud^50^ proposed two potential explanations for the maintenance of sibling species in the same geographic area without the evolution of strong premating barriers: (1) sexual reproduction may not have been frequent in nature, thus impeding strong selection for enhanced premating isolation and the fusion of insufficiently isolated populations, as we observed that the reproductive mode of some morels in nature was predominantly clonal; and (2) the sibling species may have shared only a restricted geographic contact zone (parapatry). Finally, we still have not identified the mating-type locus in morels, so the role that it plays in reproduction of different sympatric species is unknown. How these sympatric species remain reproductively isolated remains to be determined.

## Conclusions

The population genetics of two sympatric closely related species of *Morchella* was conducted for the first time in this study. Rich genetic diversity and significant population differentiation were found in *Mel-*13 and *M. eohespera*, however, *M. eohespera* possessed more variation than *Mel-*13 at the four molecular markers. We conclude that the XZRW population was probably the diversity centre of *M. eohespera* in China. Prevalent clonality, limited local recombination and potential hybridisation or horizontal gene transfer were detected in the *Morchella* taxa, but the mating systems of both species remain to be determined. Taken together, our findings suggest that the two sympatric species have a potentially divergent evolutionary history.

Genomic studies have become a research hotspot. Sequencing morel genomes will provide unprecedented insights on fruiting-related genes, the mating system and genes essential for sexual reproduction of morels.

## Methods

### Sampling

Over 1000 morels were collected from different regions of China between 2003 and 2014, covering 26 collecting sites in 10 provinces. As it is difficult to distinguish closely related morels species by morphology, We first selected 496 samples from these 26 collecting to represent each collecting site, and then screened them using sequences of four gene fragments, ITS, B2, F1 and F2. The same fragments were also used in our subsequent population genetic analyses. The screening identified 11 species from the 496 samples: *M. sexelata, M. eximia*, *M. exuberans*, *M. importuna*, *Mel-*13, *Mel-*14*, M. eximioides*, *M. eohespera*, *Mel*-21, *M. pulchella* and *Mel-*34. Based on the results combined with the phylogenetic and biogeographic results in Du *et al.*^16^, and considering that the target species should be closely related and sympatric species, more widely distributed than other species in China and represent enough samples in order to detect and compare their evolutionary histories and potential reproductive modes, we chose *Mel-*13 and *M. eohespera* for conducting comparative population genetic studies. Additionally, *Mel-*13 and *M. eohespera* were also respectively distributed in India and Europe, we tried to obtain collections from both regions for this study but failed. More effort will be made in the future for a study on the continental disjunction distribution and evolutionary history of morels. Finally, 124 collections of 11 populations representing *Mel-*13 distributed in 6 provinces (Fig. 1) and 156 collections of 14 populations representing *M. eohespera* distributed in 6 provinces (Fig. 2), covering the main distribution of both species in China, were selected for the analysis. Here, each population represented each collecting site. Maps of these collecting sites were generated using ArcView GIS 3.2 (ESRI, Redlands, CA, USA). The sample size, geographical coordinates and altitudes for each population are presented in Table 1.

### Selection of genetic markers

To develop genetic markers for our population genetic analyses, we first extracted genomic DNA from the fruiting body of isolate D139, which belonged to *Mel*-13, according to the modified CTAB method^51^. A random shotgun genomic library was constructed using genomic DNA from isolate D139 according to the methods described previously^52^.

The cloned fragments in the range 0.5–1.0 kb were amplified using vector primers and the PCR products were confirmed using agarose gel electrophoresis and then sequenced. We developed PCR primers from 15 sequences chosen from the cloned fragments using the online software Primer3 to directly amplify and sequence the DNA from four isolates: D139 (positive control), D47, D300 and D112 (the latter three strains were geographically different from D139), all of which belonged to *Mel*-13. Sequences obtained from the four strains were compared to identify single-nucleotide substitutions. Most of these primer pairs showed inconsistent PCR amplification of the samples and/or low DNA polymorphism. Finally, 3 of the 15 fragments respectively coded as B2, F1 and F2 by us, with different mutation rates and consistent PCR amplification success, never used before in morels study, were selected based on the comparison of sequences of the above four samples. The blasting results of B2, F1 and F2 fragments shown no similar results to them found in NCBI and their identity was unknown. These 3 markers together with the internal transcribed spacer (ITS) region with high nucleotide variation rates in morels, were chosen to genotype all 280 strains belonging to *Mel-*13 and *M. eohespera* from China.

### PCR amplification and sequencing

Amplifications of ITS, B2, F1 and F2 were conducted using ITS4 and ITS5^53^, B2F (5′ TGACGAGGATTGCCTTAACA 3′) and B2R (5′ AGGAGCATCATCTCCGGGTA 3′), F1F (5′ GGCTAAGATACGAGCTACGAGA 3′) and F1R (5′ ACATCAATGAGAGCCATTCG 3′) and F2F (5′ GAGCCATTCGTGCTCGTTAC 3′) and F2R (5′ ACCTGTTCGCCAGAGTTCAT 3′) for ITS, B2, F1 and F2, respectively. Each PCR reaction contained 1 μl of 20 ng/ul genomic DNA, 2.5 μl of 10 × PCR reaction buffer, 0.5 μl dNTP mix (10 mmol), 2 μl each of primer (5 umol), 1.5 μl bovine serum albumin (20 mg/ml) and 1.5 U of Taq DNA polymerase (Biomed, China). The final volume was adjusted to 25 ul with sterile distilled H_2_O. PCRs were conducted in an Applied Biosystems 2720 thermocycler (ABI, Foster City, CA), using the following cycling parameters: 94°C for 3 min, 35 cycles of 94 °C for 1 min, 50 °C (ITS and B2), 49 °C (F1) and 53 °C (F2) for 30 s, 72 °C for 1 min, followed by a final extension of 10 min at 72 °C. Amplicons were electrophoresed in 1.2% agarose in 1 × TAE, stained with GoldView™ (Guangzhou Geneshun Biotech Ltd., Guangdong, China), then photographed over an ultraviolet transilluminator. PCR products were purified using a Bioteke DNA Purification Kit (Bioteke Corporation, Beijing, China). They were used for bidirectional sequencing using the PCR primers with ABI BigDye v3.1 (Sangon Co., Ltd., Shanghai, China) and run on an ABI 3730 DNA analyser. All newly acquired sequences have been deposited as KR073664-KR073911 in GenBank.

### Data analysis

The raw sequence data of each gene fragment was edited in SeqMan (DNAStar Package, Madison, WI) and they were aligned using MUSCLE v3.8.31^54^. Aligned sequences were visually inspected and manually adjusted using BioEdit v7.0.9^55^ (http://www.mbio.ncsu.edu/bioedit/bioedit.html). As no sequence ambiguities or heterozygosity were detected, the genetic profile exhibited by each individual on the basis of four DNA fragments was treated as a haplotype on the assumption that they were haploid fungi.

### Genetic diversity and population structure

To identify the overall population structure and compare potential differences in the structure, in the following analysis we combined the four DNA fragments into one dataset respectively comprising *Mel-*13 and *M. eohespera*. Each variable nucleotide site was treated as a locus and different nucleotides at the same site were viewed as different alleles.

Indices of nucleotide diversity (π) and haplotype diversity (H_d_) were calculated for each population and each species using DNASP v5.10^56^. A Nei’s pairwise genetic distance matrix among populations was generated using GenAlEx v6.1^57^. The same software was used to perform the analysis of molecular variance (AMOVA)^58^ and Mantel test to estimate the relative contributions of geographic separation to the overall genetic variation and detect the influence of their geographic distances and altitudinal differences on their population genetic structures. The genetic relationships between geographic locations were further estimated by theta^59^ using MultiLocus v1.3^60^.

A median-joining haplotype network was constructed using NETWORK v4.5.1 (available at http://www.fluxus-engineering.com) with the MP criterion applied. The single ambiguity (closed loop) observed in the network was resolved using the frequency criterion and the geographical criterion based on coalescence theory^61^,^62^.

Neutrality tests and Pairwise mismatch distributions were implemented in ARLEQUIN v3.5.1^58^ to test the historical population dynamics. Negative statistics in neutrality tests were taken to indicate recent population expansions, whereas positive statistics indicated stationary populations. In mismatch distributions analysis, multimodal mismatch distributions and unimodal distributions are respectively expected for stationary populations and populations with recent demographic expansions^63^,^64^. The validity of the expansion model can be evaluated with the sum of squared deviations (SSD) and the raggedeness index (HRI) of observed distributions.

The spatial genetic structuring of haplotypes was analysed using spatial analysis of molecular variance analysis software, SAMOVA v1.0^65^, based on a simulated annealing approach to define groups of populations (K) that are geographically homogeneous and maximally differentiated from each other. An Φ_CT_ index^66^ of genetic differentiation among K initial groups was computed and the largest Φ_CT_ value was retained as the best population grouping.

### Clonality and recombination

The following two tests were conducted to examine evidence of recombination in an individual population and the role of sexual recombination in natural populations. First, we calculated the index of association (I_A_) and rBarD. The null hypothesis for I_A_ was that there were random associations (recombination, linkage equilibrium) between alleles at different loci. A *p* value of < 0.05 indicated that the null hypothesis should be rejected. We standardised the I_A_ value by the number of loci using the rBarD algorithm, because the value of the traditional I_A_ can be influenced by the number of loci analysed. In the second test, the proportion of pairwise loci that were phylogenetically incompatible (PI) was calculated. A lack of phylogenetic incompatibility implies asexual reproduction. The incompatibility ratio (IR), where IR = (number of incompatible pairs of sites in the test dataset)/(number of incompatible pairs of sites in a randomly shuffled dataset), can be used as a test of statistical significance^60^. The phylogenetic incompatibility and the rBarD tests were conducted using MultiLocus v1.3^60^.

## Additional Information

**Accession codes**: Nucleotide sequences associated with this article are available through GenBank (Accession nos: KR073664-KR073911).

**How to cite this article**: Du, X.-H. *et al.* High inbreeding, limited recombination and divergent evolutionary patterns between two sympatric morel species in China. *Sci. Rep.* **6**, 22434; doi: 10.1038/srep22434 (2016).

## Supplementary Material

Supplementary Information

## Figures and Tables

**Table 1:** Geographic origin, sample codes and sizes, haplotype frequencies and population genetic parameters estimated based on four molecular markers in eleven populations of *Mel*-13 and fourteen populations of *M. eohespera* included for analysis.

No.	populations locality	Code	N	Longitude	Latitude	Altitude (m)	Haplotypes (no. of individuals)	Haploid diversity(H_d_)	nucleotype diversity(π)
	*Mel*-13								
1	Zhaosu, Xinjiang	XJZS	9	80.35	42.41	2630	6[H4(2), H6(1), H7(3), H10(1), H18(1), H22(1)]	0.889	0.00083
2	Urumchi, Xinjiang	XJWLMQ	10	86.59	43.3	1490	3[H3(1), H7(8), H8(1)]	0.378	0.00021
3	Zhamashi, Qinghai	QHZM	12	100.03	38.12	2744	1[H23(12)]	0	0
4	Minhe, Qinghai	QHMH	14	102.48	36.19	1970	4[H11(7), H20(2), H23(4), H25(1)]	0.6923	0.0006
5	Babao, Qinghai	QHBB	15	100.14	38.1	2746	3[H4(7), H13(2), H20(6)]	0.648	0.00048
6	Zhugu, Qinghai	QHZG	20	102.06	37.1	3100	5[H4(11), H12(1), H20(2), H23(5), H29(1)]	0.653	0.00073
7	Fangshan, Shanxi	SXFS	10	111.21	37.49	1619	6[H1(2), H2(1), H5(3), H26(1), H27(1), H28(2)]	0.889	0.00115
8	Fu, Shaanxi	SXYA	9	109.22	35.59	938	5[H7(2), H18(1), H22(4), H23(1), H24(1)]	0.806	0.00077
9	Jiuzhaigou, Sichuan	SCJZ	10	103.55	33.15	2435	4[H19(2), H20(1), H22(6), H24(1)]	0.644	0.00034
10	Maerkang, Sichuan	SCMEK	10	102.37	31.52	3358	4[H14(1), H15(3), H16(2), H17(4)]	0.7778	0.00017
11	Laojunshan, Yunnan	YNLJS	5	99.27	27.11	2555	2[H9(1), H24(4)]	0.4	0.00045
	*M. eohespera*								
1	Zhaosu, Xinjiang	XJZS	15	80.35	42.41	2630	6 [H3(1), H8(1), H12(1), H22(8), H25(1), H30(3)]	0.705	0.00241
2	Urumchi, Xinjiang	XJWLMQ	6	86.59	43.3	1490	4[H3(1), H22(2), H30(2), H33(1)]	0.867	0.00318
3	Shangri-La, Yunnan	YNGZ	6	99.46	28.01	3128	2[H3(2), H22(4)]	0.533	0.0002
4	Ranwu, Xizang	XZRW	19	96.4	29.29	3900	10[H1(1), H6(1), H7(1), H9(1), H10(7), H11(1), H13(3), H15(2), H22(1), H23(1)]	0.854	0.00236
5	Lulang, Xizang	XZLL	5	94.44	29.46	3472	4[H2(1), H3(1), H20(1), H21(2)]	0.9	0.00242
6	Banma, Qinghai	QHBM	18	100.49	32.88	3000	7[H3(4), H9(1), H18(2),H19(8), H21(1), H30(1), H31(1)]	0.857	0.0008
7	Babao, Qinghai	QHBB	10	100.14	38.1	3476	4[H16(3), H17(5), H22(1), H24(1)]	0.771	0.00178
8	Minhe, Qinghai	QHMH	13	102.48	36.19	2746	5[H3(2), H17(5), H22(4), H26(1), H28(1)]	0.711	0.00057
9	Hongyuan, Sichuan	SCSJS	8	102.36	32	1970	3[H3(4), H5(1), H22(3)]	0.782	0.00077
10	Tianzhu, Gansu	GSTZ	15	102.84	37.24	3354	7[H3(1), H14(3), H21(2), H22(5), H24(2), H25(1), H32(1)]	0.679	0.003
11	Diebu, Gansu	GSDB	11	103.13	34.03	2387	3[H3(3), H22(7), H27(1)]	0.564	0.00044
12	Zhouqu, Gansu	GSZQ	9	104.22	33.45	1369	3[H3(1), H22(7), H29(1)]	0.417	0.00042
13	Malu, Gansu	GSML	11	103.31	34.5	2811	4[H3(2), H4(1), H5(1), H22(7)]	0.6	0.00033
14	Muer, Gansu	GSZN	10	103.29	34.34	2780	3[H3(2), H5(4), H22(4)]	0.711	0.0004

**Table 2:** Analysis of molecular variance (AMOVA) for populations of *Mel*-13 and *M. eohespera* based on data from four loci.

Source of variation	df	SS	MS	Estimated variance	*p*ercentage	AMOVA Statistics	*p* Value
*Mel*-13							
Among populations	10	714.641	71.464	5.858	49%	0.488	0.001
Within populations	113	695.327	6.153	6.153	51%		
Total	123	1409.968		12.012	100%		
*M. eohespera*							
Among groups	1	384.97	384.97	10.624	58%	0.575	0.001
Among populations	12	243.365	20.28	1.319	7%	0.647	0.001
Within populations	142	927.229	6.53	6.53	35%	0.168	0.001
Total	155	1555.564		18.472	100%		

**Table 3:** Results of the mismatch distribution analysis and neutrality tests of *Mel*-13 and *M. eohespera*.

S*p*ecies	SSD	*p*-value	R	*p*-value	Tajima’s D	*p*-value	Fu’s FS	*p*-value
*Mel*-13	0.108	0.206	0.227	0.37	0.182	0.62	1.403	NA
*M. eohespera*	0.123	0.235	0.182	0.514	0.139	0.560	0.475	0.531

**Table 4:** Multilocus linkage disequilibrium analyses for each gene fragment respectively in the total samples of *Mel*-13 and *M. eohespera*.

species	Gene fragment	PrC (*p*value)	I_A_ (*p* value)	rBarD (*p*value)
*Mel*-13 (124 samples)	B2	NA	0.000(*p* = 1)	NA
	F1	0.800(*p *< 0.001)	0.443(*p *< 0.001)	0.100(*p *< 0.001)
	F2	1.000(*p *< 0.001)	0.792(*p *< 0.001)	0.301(*p *< 0.001)
	ITS	1.00000(*p *< 0.001)	4.261(*p *< 0.001)	0.508 (*p *< 0.001)
*M. eohespera* (156 samples)	B2	1.000(*p* = 0.003)	0.950(*p *< 0.001)	0.388(*p *< 0.001)
	F1	1.000(p < 0.001)	2.312(p < 0.001)	0.336(*p *< 0.001)
	F2	1.000(*p *< 0.001)	2.396(*p *< 0.001)	0.300(*p *< 0.001)
	ITS	1.000(*p *< 0.001)	-0.015(*p* = 0.528)	-0.001(*p* = 0.528)

**Table 5:** Multilocus linkage disequilibrium analyses for the combined data with four DNA fragments as four loci in samples of *Mel*-13 and *M. eohespera*.

Sample set	Sample size	prC (p value)	I_A_ (p value)	rBarD (p value)
*Mel*-13				
Total samples	124	0.500(*p* = 1)	0.544(*p* < 0.001)	0.208(*p* < 0.001)
XJZS	9	1.000(*p* = 0.434)	0.392(*p* = 0.11)	0.198(*p* = 0.11)
XJWLMQ	10	1.000(*p* = 1)	0.548 (*p* = 0.183)	0.557(*p* = 0.183)
QHZM	12	1.000(*p* = 1)	NA	NA
QHMH	14	1.000(*p* < 0.001)	1.377(*p* < 0.001)	0.688(*p* < 0.001)
QHBB	15	1.000(*p* = 0.026)	2.990(*p* < 0.001)	0.996(*p* < 0.001)
QHZG	20	1.000(*p* < 0.001)	1.252(*p* < 0.001)	0.626(*p* < 0.001)
SXFS	10	0.833(p = 1)	0.129(*p* = 0.226)	0.065(*p* = 0.226)
SXYA	9	1.000(*p* = 0.279)	0.154(*p* = 0.321)	0.077(*p* = 0.321)
SCJZ	10	1.000(*p* = 1)	0.270(*p* = 0.394)	0.275(*p* = 0.394)
SCMEK	10	1.000(*p* = 0.134)	0.562(*p* = 0.009)	0.571(*p* = 0.009)
YNLJS	5	1.000(*p* = 1)	1.000(*p* = 0.2)	1.000(*p* = 0.2)
*M. eohespera*				
Total samples	156	0.000(*p* = 1)	1.283(*p* < 0.001)	0.427(*p* < 0.001)
XJZS	15	1.000(*p* < 0.001)	2.261(*p* < 0.001)	0.754(*p* < 0.001)
XJWLMQ	6	1.000(*p* = 0.003)	2.297(*p* < 0.001)	0.787(*p* < 0.001)
YNGZ	6	1.000(*p* = 1)	0.000(*p* = 1)	NA
XZRW	19	0.833(*p* = 0.023)	1.285(*p* < 0.001)	0.429(*p* < 0.001)
XZLL	5	1.000(p = 1)	1.356(p = 0.004)	0.453(*p* = 0.004)
QHBM	18	1.000(*p* < 0.001)	1.676(*p* < 0.001)	0.560(*p* < 0.001)
QHBB	10	1.000(*p* = 1)	0.843(*p* = 0.007)	0.426(*p* = 0.007)
QHMH	13	1.000(*p* < 0.002)	0.789(*p* < 0.001)	0.394(*p* < 0.001)
SCSJS	8	1.000(*p* = 1)	−2.22045e−16(*p* = 1)	NA
GSTZ	15	1.000(*p* = 0.002)	0.560(*p* = 0.001)	0.301(*p* = 0.001)
GSDB	11	1.000(*p* = 1)	2.262(*p* < 0.001)	0.754(*p* < 0.001)
GSZQ	9	1.000(*p* = 1)	2.382(*p* = 0.004)	0.800(*p* = 0.004)
GSML	11	1.000(*p* = 1)	0.000(*p* = 1)	NA
GSZN	10	1.000(*p* = 1)	0.000(*p* < 0.001)	NA
