# Supplementary material for: High inbreeding, limited recombination and divergent evolutionary patterns between two sympatric morel species in China
Source: Sci Rep. 2016 Mar 1;6:22434. doi: 10.1038/srep22434 (PMC4772476; doi:10.1038/srep22434)
Supplement: Supplementary Information [file srep22434-s1.doc]

**High inbreeding, limited recombination and divergent evolutionary patterns between two sympatric morel species in China**

Xi-Hui Du^1#^ Qi Zhao^1#^ Jianping Xu^2^  Zhu L. Yang^1^*

^1^Key Laboratory for Plant Diversity and Biogeography of East Asia, Kunming Institute of Botany, Chinese Academy of Sciences, Kunming, Yunnan 650201, China

^2^Department of Biology, McMaster University, Hamilton, ON L8S 4K1, Canada

^#^These authors contributed to the work equally and should be regarded as co-first authors.

*Corresponding author: Dr. Zhu L. Yang

Address: 132#, Lanhei Road, Panlong District, Kunming, China

E-mail: [fungi@mail.kib.ac.cn](mailto:fungi@mail.kib.ac.cn)

Table S1 *Mel*-13 pairwise population matrix of Nei genetic distance

| XJZS | XJWLMQ | QHZM | QHMH | QHBB | QHZG | SXFS | SXYA | SCJZ | SCMEK | YNLJS |  |
| --- | --- | --- | --- | --- | --- | --- | --- | --- | --- | --- | --- |
| 0.000 |  |  |  |  |  |  |  |  |  |  | XJZS |
| 0.018 | 0.000 |  |  |  |  |  |  |  |  |  | XJWLMQ |
| 0.323 | 0.538 | 0.000 |  |  |  |  |  |  |  |  | QHZM |
| 0.210 | 0.320 | 0.150 | 0.000 |  |  |  |  |  |  |  | QHMH |
| 0.049 | 0.104 | 0.226 | 0.173 | 0.000 |  |  |  |  |  |  | QHBB |
| 0.042 | 0.098 | 0.196 | 0.132 | 0.009 | 0.000 |  |  |  |  |  | QHZG |
| 0.119 | 0.213 | 0.157 | 0.226 | 0.056 | 0.075 | 0.000 |  |  |  |  | SXFS |
| 0.107 | 0.212 | 0.097 | 0.188 | 0.091 | 0.097 | 0.043 | 0.000 |  |  |  | SXYA |
| 0.201 | 0.339 | 0.102 | 0.262 | 0.153 | 0.174 | 0.059 | 0.015 | 0.000 |  |  | SCJZ |
| 0.245 | 0.251 | 0.558 | 0.180 | 0.346 | 0.290 | 0.550 | 0.463 | 0.663 | 0.000 |  | SCMEK |
| 0.196 | 0.338 | 0.042 | 0.146 | 0.207 | 0.170 | 0.152 | 0.056 | 0.086 | 0.384 | 0.000 | YNLJS |

Table S2 *Mel*-19 pairwise population matrix of Nei genetic distance

| XJZS | XJWLMQ | YNGZ | XZRW | XZLL | GSTZ | QHBM | QHBB | QHMH | SCSJS | GSDB | GSZQ | GSML | GSZN |  |
| --- | --- | --- | --- | --- | --- | --- | --- | --- | --- | --- | --- | --- | --- | --- |
| 0.000 |  |  |  |  |  |  |  |  |  |  |  |  |  | XJZS |
| 0.023 | 0.000 |  |  |  |  |  |  |  |  |  |  |  |  | XJWLMQ |
| 0.027 | 0.092 | 0.000 |  |  |  |  |  |  |  |  |  |  |  | YNGZ |
| 0.100 | 0.041 | 0.227 | 0.000 |  |  |  |  |  |  |  |  |  |  | XZRW |
| 0.026 | 0.067 | 0.023 | 0.167 | 0.000 |  |  |  |  |  |  |  |  |  | XZLL |
| 0.033 | 0.105 | 0.013 | 0.234 | 0.032 | 0.000 |  |  |  |  |  |  |  |  | GSTZ |
| 0.023 | 0.072 | 0.019 | 0.180 | 0.027 | 0.023 | 0.000 |  |  |  |  |  |  |  | QHBM |
| 0.053 | 0.125 | 0.028 | 0.254 | 0.057 | 0.033 | 0.048 | 0.000 |  |  |  |  |  |  | QHBB |
| 0.035 | 0.104 | 0.010 | 0.235 | 0.036 | 0.019 | 0.030 | 0.006 | 0.000 |  |  |  |  |  | QHMH |
| 0.035 | 0.099 | 0.003 | 0.239 | 0.025 | 0.020 | 0.025 | 0.036 | 0.016 | 0.000 |  |  |  |  | SCSJS |
| 0.025 | 0.092 | 0.001 | 0.224 | 0.024 | 0.012 | 0.020 | 0.027 | 0.009 | 0.005 | 0.000 |  |  |  | GSDB |
| 0.024 | 0.091 | 0.003 | 0.221 | 0.026 | 0.011 | 0.020 | 0.026 | 0.009 | 0.009 | 0.001 | 0.000 |  |  | GSZQ |
| 0.028 | 0.094 | 0.000 | 0.230 | 0.024 | 0.014 | 0.021 | 0.029 | 0.011 | 0.002 | 0.002 | 0.003 | 0.000 |  | GSML |
| 0.039 | 0.104 | 0.006 | 0.244 | 0.030 | 0.024 | 0.028 | 0.040 | 0.019 | 0.002 | 0.008 | 0.012 | 0.004 | 0.000 | GSZN |


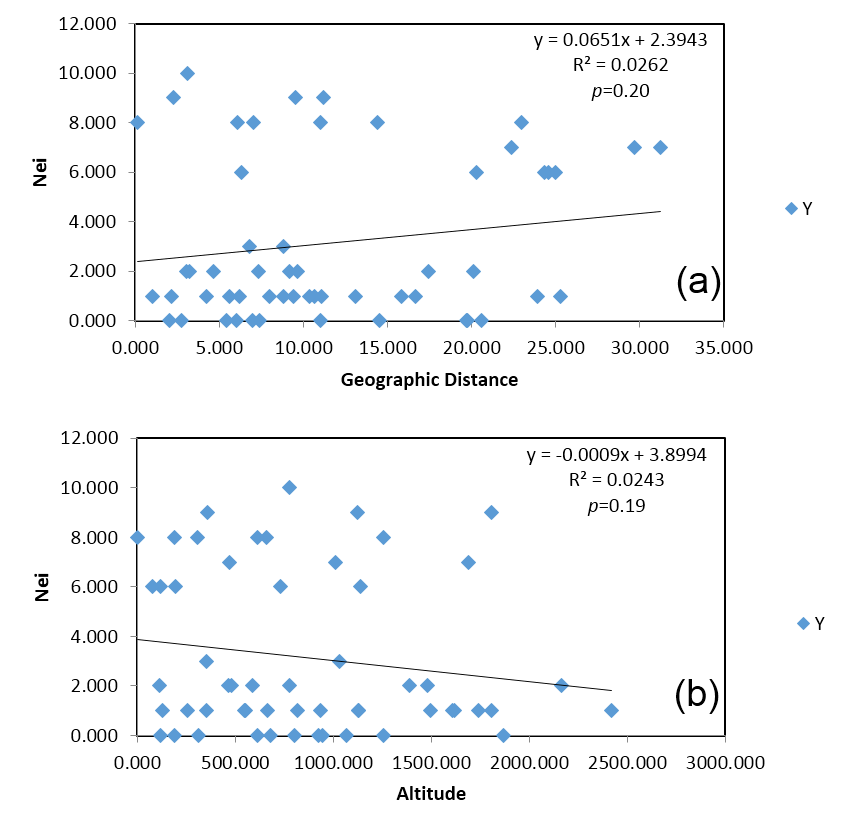


Figure S1 Results from two Mantel tests between genetic differences and geographic distances among populations of *Mel*-13. (a) A Mantel test between Nei’s genetic distance and the two-dimensional geographical distances (based on longitudinal and latitudinal coordinates) among populations. (b) A Mantel test between Nei’s genetic distance and altitudinal differences between populations.


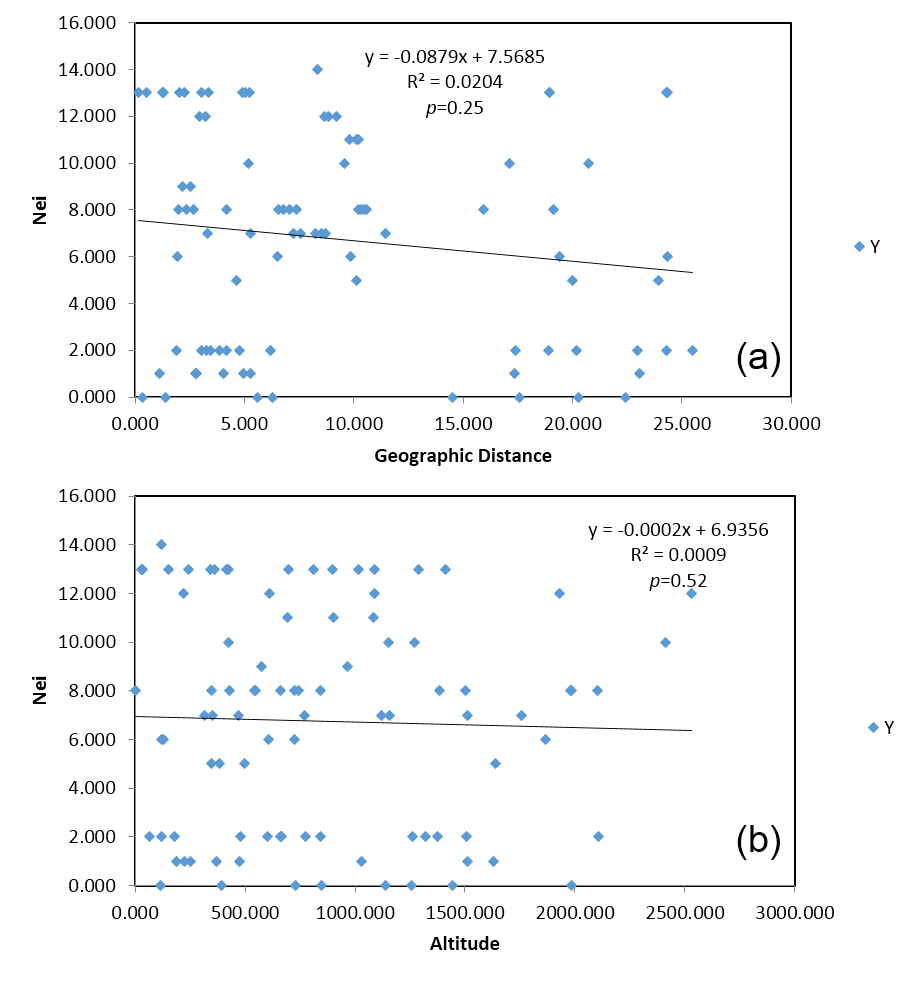


Figure S2 Results from two Mantel tests between genetic differences and geographic distances among populations of *Mel*-19. (a) A Mantel test between Nei’s genetic distance and the two-dimensional geographical distances (based on longitudinal and latitudinal coordinates) among populations. (b) A Mantel test between Nei’s genetic distance and altitudinal differences between populations.


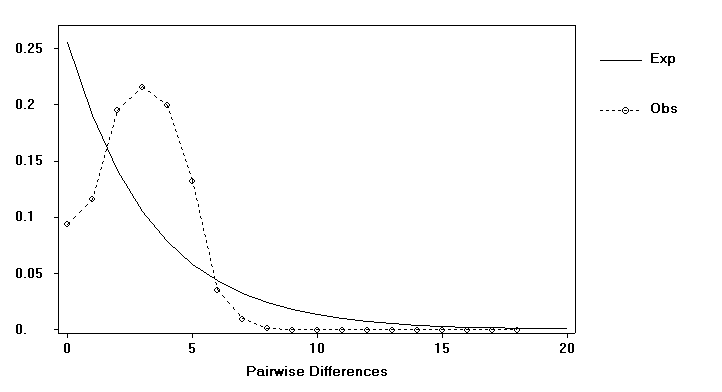


Figure S3 Mismatch distributions of haplotypes based on pairwise sequence differences as a function of the frequencies of occurrence for *Mel*-13. Along the x axis are the numbers of pairwise nucleotide differences between haplotypes and their frequencies are along the y axis.


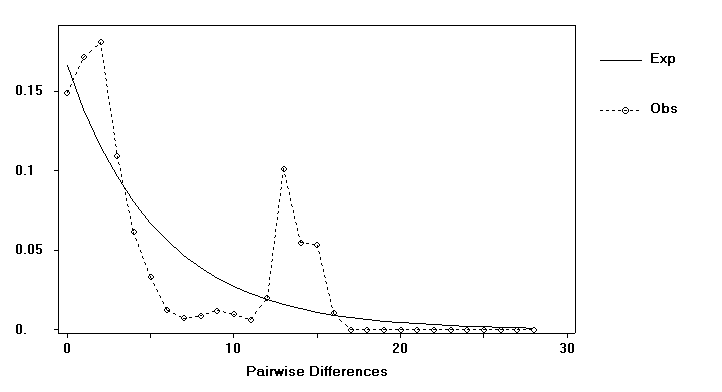


Figure S4 Mismatch distributions of haplotypes based on pairwise sequence differences as a function of the frequencies of occurrence for *Mel*-19. Along the x axis are the numbers of pairwise nucleotide differences between haplotypes and their frequencies are along the y axis.
